# 

**Published:** 1841-09

**Authors:** 


### AMERICAN JOURNAL

OF

### DENTAL SCIENCE.

THE

### AMERICAN JOURNAL

OF

### Dental Science.

77

PUBLISHED UNDER THE AUSPICES OF THE

American Society of Dental Surgeons.

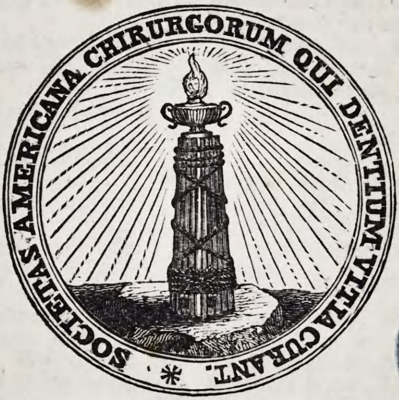

SURGEON GEN'LS OFFICE
LIBRARY.
13370

VOLUME II.—1841-2.

EDITED BY

CHAPIN A. HARRIS, M. D., D. D. S.

AND

SOLYMAN BROWN, M. D., D. D. S.

NEW YORK:—GEORGE ADLARD,

BALTIMORE:—ARMSTRONG & BERRY,

LONDON:—SAMUEL HIGHLEY.

1841 —1842.

W
1
AM 45 M

WOODS & CRANE, PRINTERS,
*Baltimore.*

### INDEX.

PAGE.

Address, Opening, by Prof. H. H. Hayden, . . . . . 1
American Society of Dental Surgeons, proceedings of 1841-42, 134–256
American Journal and Library of Dental Science, . . . . 145
America, progress of dental science in, . . . . . 160
Alveolar exostosis, case of, by S. M. Shepherd, . . . . 193
Anatomy and physiology of the teeth, &c. &c. by D. W. Jobson, M. R. C. S. &c. . . 195
An appeal to parliament, &c. on the present state of dental surgery. By G. Waite, Esq. 206
Advice on the care of the teeth. By E. Saunders, . . . . 207
Anomalous Dentition, . . . 208
Animal magnetism, . . . . 212
Anchylosis of the lower jaw with the temporal bone, . . . 260
Answers to questions of a correspondent, . . . . . 264
Acknowledgment, . . . 263
Annual dues, . . . . . 159
Antrum, maxillary, observation on abscess of the, by S. P. Hullihen, . . . . . 179
Antrum, maxillary, muco-purulent secretion of the, by S. P. Hullihen, . . . . . 253
Arsenious acid for destroying nerves of decayed teeth, by W. E. Ide, M. D. . . . 247
Agent, Philadelphia, . . . 263
Alveolar Abscess. By I. I. Greenwood, M. D. &c. . 291

PAGE.

Ashburner, J. M. D. on dentition, 294
Amalgam of mercury and silver, salivation from, &c. &c. 301
American Journal and Library of dental science, vol. 2. 302

B

Brown, Mr. C. letter from, 136–159
Baume, prof. work on dentition, 158
Brewster, Geo. G. on galvanism, 188
Baltimore College of Dental Surgery, . . . . 207–261
Bibliography, . . . . . 256
Burton, Henry, M. D. &c. on the effects of absorption of lead upon the gums, . 140
Brown, Solyman, M. D., D. D. S. on mechanical dentistry, 161–231–265

C

Conjoined suppuration of the gums, by H. H. Hayden, M. D. 214
Clerical encouragement of quackery, . . . . . 262
Calculus salivary. By C. A. Harris, M. D. . . . . 99
Characteristics of the teeth, 65
of the gums, . . . 84
of the salivary calculus, 99
of the fluids of the mouth, 108
of the lips, . . . . 113
of the tongue, . . . 115

D

Dissertation on the Mouth and Teeth, by L. S. Parmly, M. D. 28

PAGE.

Dissertation on professional eminence, by S. Brown, M. D. 120
Dental Surgeons, American Society of, . . . 134–256
Dental Surgeons, officers of the American Society of, . . 134
Dental Science, American Journal and Library of . . . 145
Development, Structure and disease of the teeth. By A. Nasmyth, F. L. S. F. G. S. 146–156
Dental Science, progress of in America, . . . 160–299
Dentistry, mechanical, a treatise on, by S. Brown, M. D. 161–231–265
Dental art, statistics of in Paris in 1828, . . . . . 192
Dental art, complete treatise on, 298
Dentist, every man his own, &c. By J. Scott, . . . 201
Dentistry, surgical, operative and mechanical. By Charles De Loud, . . . . . 156
Dental works in progress, 208
Dental bone, on the vascularity of, by C. A. Harris, M. D. 252
Diseases of the gums, . . 263
Diseases of the gums, conjoined suppuration of, by H. H. Hayden, M. D. . . . . 214
Dental and Surgical Instruments, 263
Diseases of the maxillary sinuses. By W. W. H. Thackston, D. D. S. . . . . . 279
Dentition and some coincident disorders. By J. Ashburner, M. D. . . . . . 293
Dental surgery, institutes of, by J. C. McCabe, . . . 297

E.

Editorship, change in the, . 145
Embellishments, . . . . 160
Exostosis, Alveolar, by S. M. Shepherd, . . . . . 193
Encouragement of quackery, clerical, . . . . . . 262
Essays, Popular, on Dental Surgery. . . . . . 159

F.

Fluid of the Mouth, characteristics of, by C. A. Harris, M. D. 108

PAGE.

Foster, J. H., M. D. letter from, 130

G

Gums, characteristics of, by C. A. Harris, M. D. . . . . 84
Gums, their structure, growth, &c. by G. Waite, M. R. C. S. 206
Gums, conjoined suppuration of the, by H. H. Hayden, M. D. 214
Gums, Disease of the, . 263
Gums, Koecker, Dr. on, . 298
Gums, remarkable effects upon the human, produced by the absorption of Lead, . 140
Greenwood, Dr. Isaac I. letter from, &c. . . . . . 138
Greenwood, Dr. Isaac I. letter from, &c. . . . . . 177
Greenwood, Dr. Isaac I. on Alveolar Abscess, .. . . 291
Goodsir, John R. on the Pulps and Sacs of the human teeth, 156–202
Galvanism, by G. G. Brewster, 188
Gray, John, on Preservation of the Teeth, . . . . 207
Gardette, Dr. E. B. and the American Society of Dental Surgeons, . . . 209

H.

Hayden, Professor H. H. Opening address, &c. . . 1–157
Hayden, Professor H. H. on the Gums, &c. . . . . . 213
Harris, Professor C. A. on the Teeth, Gums, &c. . . 39
Harris, Prof. C. A. on Ozœna, 184
Harris, C. A., M. D., on the Vascularity of Dental Bone, 252
Human Teeth, practical treatise on, by W. Robertson, . 150
Human Teeth, popular treatise on, J. L. Murphy. . . . 155
Human Teeth, Pulps and Sacs of, by J. Goodsir, Jr. 156–202
Health, Guardian of, . . 157
Hullihen, S. P. Observations on Abscess of the Antrum Maxillary, . . . . . . 179
Hullihen, S. P. on Muco-purulent secretion of the antrum maxillary, . . . . 253

PAGE.

Hemorrhage from the extraction of a tooth, . . . . 304

I.

Inquiry, physiological and pathological, on the teeth, gums, &c. by C. A. Harris, M. D. &c. 39
Ide, W. E., M. D. on the use of arsenious acid for destroying nerves of decayed teeth, . 247
Instruments, dental and surgical, 263

J.

Journal, American and Library, of Dental Science, . . 145
Jobson, D. W., M. R. C. S. &c. &c. on the anatomy and physiology of the teeth, &c. . 195

K.

Koecker, Dr. on the diseases of the gums, . . . 299

L.

Lips, characteristics of, . . 113
Letter, from J. H. Foster, . 130
Letter from Mr. C. Brown, . 136
Letter from Dr. I. I. Greenwood, 138–177
Letter from J. E. Snodgrass, M. D. . . . . . . 141
Lead, effect upon the human gums produced by absorption of, 140
Lintot, Wm. on the structure, economy and pathology of the teeth, . . . . . . 207
Lefoulon, Manuel, Pratique de l'art du dentiste. . . . 207

M.

Meeting in Boston, . . . 302
Mechanical Dentistry, by Solyman Brown, M. D., D. D. S. 161–231–265
Mechanical Dentistry, surgical and operative, . . . . 156
Mouth and teeth, management of, by L. S. Parmly, M. D. D. D. S. . . . , . 28
Materials for plugging teeth, . 158

PAGE.

Members, officers, &c. of American Society of Dental Surgeons, 134
Maxillary antrum, observations on, by S. P. Hullihen, . 179
Maxillary antrum, muco-purulent secretion of, by S. P. Hullihen, . . . . . 253
Maxillary antrum, disease of, by W. W. H. Thackston, D. D S. 279
Medical Almanac for 1842, 145
Manuel pratique de l'art du dentiste, par Lefoulon, . . 207
Magnetism, animal, . . 212
Maxillary bone, removal of the superior, . . . . . 251
Maxilla, lower, anchylosis of the with the temporal bone, . 260
Married, . . . . . 264
Murphy, J. L. treatise on the teeth, . . . . . . 155
McCabe, J. D. . . . 297

N.

Nasmyth, Alexander, F. L. S., F. G. S. researches on the teeth, . . . - 146–204

O.

Opening address, by H. H. Hayden, M. D., D. D. S. . . 1
Officers and members, . . 134
Owen, Richard, F. R. S. &c. odontography, . . . . 156
Origin and development of the pulps and sacs of the teeth, by J. Goodsir, jr. . . 156–202
Omission, . . . . . . . 157
Ozœna, by C. A. Harris, M. D. 184
Obituary, . . . . . 264

P.

Professional eminence, by Solyman Brown, M. D. . . 120
Preservation of the teeth, 158–207
Popular essays on dental surgery, 159
Phenomenon, singular, . 160
Progress of dental science in America, . . . 160–299
Porcelain teeth, . . . 258
Philadelphia agent, . . 263
Parmly, L. S., M. D. &c. on the mouth and teeth, . . . 28

PAGE.

Plugging teeth, . . . . 210

Q

Quackery, clerical encouragement of, . . . . . 262
Questions, answers to, of a correspondent, . . . 264

R.

Researches on the development &c. of the teeth, by A. Nasmyth, F. L. S. &c. . 146–204
Remarkable effects of the absorption of lead up the gums, by H. Burton, M. D. . . 140
Robertson, William, on the teeth, 150

S.

Society of Dental Surgeons, American, . . . 134–209–256
Snodgrass, J. E., M. D. letter from, . . . . . . 141
Science, Dental, American Journal and Library of, &c. . 145
Surgical, operative and mechanical dentistry, by C. De Loud, 156
Singular Phenomenon, . 160
Science, Dental, progress of in America, . . . . 160–299
Shepherd, S. M. dentist, on alveolar exostosis, . . . 193
Scott, Joseph, on the teeth, . 201
Saunders, Edward, advice on the care of the teeth, . . 207
Sinuses maxillary, diseases of, by W. W. H. Thackston, D. D. S. . . . . . 279
Schange, J. M. A. Precis sur le redressement des dents, 298
Statistics of the dental art in Paris in 1828, . . . . 192
Salivary calculus, physical characteristics of, . . . 99

PAGE.

T.

Teeth and mouth, management of, by L S. Parmly, M. D. &c. . . . . . 28
Teeth, characteristics of, . 65
Development, structure, and diseases of, by A. Nasmyth, F. L. S. &c. 146–204
Practical treatise on, by W. Robertson, . . 150
Popular treatise on, by J. L. Murphy, . . . 155
Origin and development of the pulps and sacs of, 156–202
Development and structure of, . . . . 156
Treatment of the primary, 156
Plugging of, . . 156
Preservation of, . 158–207
Materials for plugging 158
Economy and pathology of, 207
Diseases and treatment of, 207–296
Advice on the care of, . 207
Decayed, arsenious acid, for destroying nerve in, . 247
Porcelain, . . . 258
Thackston, W. W. H., D. D. S. on diseases of the maxillary sinuses, . . . . . 279
Tongue, physical characteristics of, . . . . . . 115

V.

Vascularity of dental bone, . 252

W.

Work, Professor Baume's, . 158
Waite, George, Esq. an appeal by, . . . . . . 206
Waite, George, on the gums, 206
Wardroper, W. on the teeth, . 207
Wonderful discovery, . 259
Whitewood, Mr. . . . 208

AMERICAN JOURNAL AND LIBRARY OF DENTAL SCIENCE.

*Change in the New York Editorship.*—With the transfer of our Journal from the hands of the original proprietors, to the American Society of Dental Surgeons, there has been a change, as our readers will perceive, in the New York editorship. Dr. E. Parmly, to whose zeal and ability they have been so much indebted, declining on account of his arduous professional, and numerous other duties, to be a candidate for the editorship, Dr. Solyman Brown was chosen by the society to supply his place.

We regret as much as any of our readers can, the loss of Dr. Parmly's valuable services, but we are happy to know that his place has been ably filled, and that his zeal in the cause of the profession is undiminished,—that his steady and efficient aid will be given to the Journal as cheerfully, though perhaps less publicly than heretofore.

In retiring from the editorship of the Journal, Dr. Parmly takes with him our grateful remembrance, and our best wishes. May he live to see the cause which lays so near his heart, prosper far beyond his most sanguine expectations; and when he has passed away, may the dental profession, rescued from degradation, and elevated to honour and acknowledged importance, be his enduring monument.—*Balt. Ed.*

*Medical Almanac, for* 1842.—Gentlemen preparing articles for the next volume, the 4th in the series, are requested to transmit them to the address of the editor of this Journal, as soon as it will suit their convenience. Medical statistics, in the United States and the British American Provinces, are especially desired—together with accounts of all new medical associations, the names of their officers, and all other useful information concerning them. Full and accurate accounts of medical schools, hospitals, infirmaries and dispensaries, as in past years, are requested from authentic sources. Any communications calculated to make this annual increasingly useful to the profession throughout the whole country, will be gratefully acknowledged by the editor.—*Boston Medical and Surgical Journal.*

*Odontography;* or, a treatise on the Comparative Anatomy of the Teeth, the Physiological Relations, Mode of Development, and Microscopic Structure, in the vertebrate animals, illustrated by upwards of 150 plates. By Richard Owen, F. R. S., &c. &c., London, 1840–41. We have received the first and second parts of this most splendid and magnificent work. Our limits at present precludes any further notice of it. We shall notice it, however, at some length, when it is completed. The third and last part is promised in 1842.—*Balt. Ed.*

*On the Origin and Development of the Pulps and Sacs of the Human Teeth.* By John Goodsir, Jr., *Surgeon, Anstruther, Fifeshire.*—The above is the title of a very excellent paper of thirty-eight pages, published in the *Edinburg Medical and Surgical Journal.* A synopsis of the views of the author, we shall endeavour to present to our readers in a future number.

*Development and Structure of the Teeth.*—We learn from the last number of the "Boston Medical and Surgical Journal," that three memoirs, read at a meeting of the British Association, on the development and structure of the Teeth and Epithelium, by Aexander Nasmyth, F. L. S., F. G. S. &c. has been published.

These have been long anxiously looked for, and we are gratified to learn that they have at last appeared. We hope soon to be able to give a more extended notice of Mr. Nasmyth's views upon this highly interesting subject.

*Surgical Operative and Mechanical Dentistry,* &c. &c. by Chas. De Loud, Surgeon Dentist, &c.; London, 1840; pp. 196, 8vo. The above treatise was received from the author, by the Baltimore editor, for the Baltimore College of Dental Surgery.

*Treatment of the Primary Teeth.*—M. Delmond, has recently published a paper in a French journal, as we learn from "The American Journal of Medical Sciences," on this subject.

*Plugging Teeth.*—We learn from the excellent and ably conducted Medical Journal, alluded to in the preceding notice, that M. O. Taveau, has published in the French Lancet, some remarks upon this operation. We may notice them at length in a subsequent number.

*The Guardian of Health.*—As the relation of one of the editors of the American Journal and Library of Dental Science, to the above named periodical rendered it improper for us to notice it, we had determined to pass it by in silence, when we received the Boston Medical and Surgical Journal, containing the annexed remarks upon it.

The known character of this Journal gives us courage to lay before our readers its words of commendation. From a less distinguished source we should have hesitated to do so.

"*Guardian of Health.*—Thomas E. Bond, Jr., M. D. and Chapin A. Harris, M. D., of Baltimore, are the joint editors of an interesting monthly periodical, devoted to domestic hygiene, with the above title, a specimen of which, comprising Nos. 1, 2 and 3, is before us. In every family, whether at the south or north, this publication would be prized if there was a single ray of intelligence in the household. The article entitled '*Remedies in cases of Poisons and Accidents,*' printed on a card, and nailed up in every dwelling throughout the entire country, would save many lives which are annually sacrificed as martyrs to ignorance. The paper on croup is worth committing to memory by all mothers. In a word, the plan and whole execution of this new journal is unexceptionable, and we shall be gratified to aid in extending its circulation as widely as its merits deserve."

At the request of friends in New York, the 'Guardian of Health,' will hereafter be printed in newspaper form, and will contain such advertisements as may be proper to a journal devoted to the preservation of health. It will also be furnished in pamphlet form to all who desire it. Persons ordering it, therefore, will be particular to mention the form in which they wish to have it sent to them.

